# Continuity and care coordination of primary health care: a scoping review

**DOI:** 10.1186/s12913-023-09718-8

**Published:** 2023-07-13

**Authors:** Resham Khatri, Aklilu Endalamaw, Daniel Erku, Eskinder Wolka, Frehiwot Nigatu, Anteneh Zewdie, Yibeltal Assefa

**Affiliations:** 1 School of Public Health, the University of Queensland, Brisbane, Australia; 2 Health Social Science and Development Research Institute, Kathmandu, Nepal; 3 College of Medicine and Health Sciences, Bahir Dar University, Bahir Dar, Ethiopia; 4 Centre for Applied Health Economics, School of Medicine, Griffith University, Mount Gravatt, Australia; 5 Menzies Health Institute Queensland, Griffith University, Mount Gravatt, Australia; 6 International Institute for Primary Health Care-Ethiopia, Addis Ababa, Ethiopia

**Keywords:** Continuity of care, Care coordination, Care continuity, Primary health care, Primary care

## Abstract

**Background:**

Healthcare coordination and continuity of care conceptualize all care providers and organizations involved in health care to ensure the right care at the right time. However, systematic evidence synthesis is lacking in the care coordination of health services. This scoping review synthesizes evidence on different levels of care coordination of primary health care (PHC) and primary care.

**Methods:**

We conducted a scoping review of published evidence on healthcare coordination. PubMed, Scopus, Embase, CINAHL, Cochrane, PsycINFO, Web of Science and Google Scholar were searched until 30 November 2022 for studies that describe care coordination/continuity of care in PHC and primary care. We followed the Preferred Reporting Items for Systematic Reviews and Meta-Analyses Extension for Scoping Reviews (PRISMA-ScR) guidelines to select studies. We analysed data using a thematic analysis approach and explained themes adopting a multilevel (individual, organizational, and system) analytical framework.

**Results:**

A total of 56 studies were included in the review. Most studies were from upper-middle-income or high-income countries, primarily focusing on continuity/care coordination in primary care. Ten themes were identified in care coordination in PHC/primary care. Four themes under care coordination at the individual level were the continuity of services, linkage at different stages of health conditions (from health promotion to rehabilitation), health care from a life-course (conception to elderly), and care coordination of health services at places (family to hospitals). Five themes under organizational level care coordination included interprofessional, multidisciplinary services, community collaboration, integrated care, and information in care coordination. Finally, a theme under system-level care coordination was related to service management involving multisectoral coordination within and beyond health systems.

**Conclusions:**

Continuity and coordination of care involve healthcare provisions from family to health facility throughout the life-course to provide a range of services. Several issues could influence multilevel care coordination, including at the individual (services or users), organizational (providers), and system (departments and sectors) levels. Health systems should focus on care coordination, ensuring types of care per the healthcare needs at different stages of health conditions by a multidisciplinary team. Coordinating multiple technical and supporting stakeholders and sectors within and beyond health sector is also vital for the continuity of care especially in resource-limited health systems and settings.

**Supplementary Information:**

The online version contains supplementary material available at 10.1186/s12913-023-09718-8.

## Introduction

Understanding continuity and coordination of care is vital for delivering and utilizing primary health care (PHC) (PHC covers the principle of equity, intersectorality, community participation, and affordable/appropriate care) and primary care (PC) (primary level of care where people make first contact with the health care delivery systems). The concept varies care continuity, coordination, integration, patient-centred care, continuous, cohesive and consistent care for illnesses [1]. Care coordination ensures that all providers and organizations involved in health care provide the right care at the right time, involving a people-centric approach and ensuring clients are duly informed of their preferences [2, 3]. This concept also refers to healthcare components from various sources, supports, patients, types of care, service levels, and time dimensions [4] or perspectives at the individual, organizational or system levels [5, 6].

Care coordination ensures people-centred care, covering discrete healthcare events experienced by people as coherent and interconnected over time, consistent with their health needs and preferences, bringing and meeting health needs and ensuring integrated care [7]. Furthermore, care coordination refers to interprofessional care, patient-centred care, self-management support, prevention, screening, primary care, and treatment of illnesses [8, 9]. Other features of health care coordination include multidisciplinary services, establishing cooperative and ongoing relationships, and delivering multiple health services (e.g., case management of all stages of disease), especially for people with multiple morbidities [7, 10].

Moreover, healthcare coordination or continuity care can be explained as informational continuity (communication among providers), relational (provider-patient relationship, team-driven continuity), and management continuity (activities for systems and service organizations) [11, 12]. This informational and relational care coordination occurs at the individual and organisational level for relationship, communication and cooperation between providers and users [1, 13]. The level of stakeholders engagement in care coordination/continuity of care depends on the hierarchical and interdependent relationship in the context of time and setting of health systems [6]. Care coordination within the organisation and systems supports planning and managing integrated health services by involving interdisciplinary or interprofessional teams [14, 15]. Shared decision-making is essential in policy, practice, and research that could influence people-centred integrated public health and primary care [7].

The current body of literature focuses on concepts and empirical studies on care coordination and continuity of care. However, systematic synthesis of available evidence in care coordination is vital to identify issues, challenges, and approaches for delivering and utilising health services. Nonetheless, there are limited studies that synthesised evidence in the health care coordination in PHC and primary care. This review synthesises evidence on care coordination in providing and delivering PHC and primary care.

## Methods

### Study design

This study is a systematic scoping review of studies on continuity of care/care coordination in PHC and primary care. We employed Preferred Reporting Items for Systematic Reviews and Meta-Analyses Extension for Scoping Reviews (PRISMA-ScR) guidelines (Supplementary information, Table S1) [16].

### Identifying research question

The following questions guided the scoping review: 1) How does care coordination occur in PHC and primary care service delivery? 2) What factors contribute to coordination/continuity of care of PHC and primary care services?

### Search strategy

We searched seven databases (PubMed, Scopus, Embase, CINAHL, Cochrane, PsycINFO, and Web of Science). Complementary Google Scholar followed these searches for citation searches of included studies. The search strategy was built on several search terms under two key themes: coordination (coordination or collaboration or cooperation or “intersectoral coordination” or “continuum of care” or “service coordination” or “care continuum” or “team approach” or “service referral” or “service linkage” or continuity); and PHC (“primary health care” or “primary care” or “health care”) on each database. Boolean operators and truncations varied depending on the database.

### Eligibility criteria

The search included articles published in English until 30 November 2022. No country-related limitations were applied. We included all types of studies that dealt with healthcare coordination regardless of the design of the studies.

### Study selection

The first author screened abstracts of studies retrieved from the search. The second author further assessed the screening. The screening process was followed by a full-text reading initially by the first author and assessed by the second and third authors. Any disagreements were resolved by discussion. We included studies in the review if the data/findings contributed to the research questions. We followed the standard scoping review PRISMA-ScR guideline [16, 17] and previous scoping reviews [18, 19]. The included studies are based on the findings and interpretation considering the quality of the included studies rather than as inclusion criteria itself [20, 21]. To effectively answer research questions, we adopted the Joanna Briggs Institute framework covering each study's population (services users and providers), concept (care coordination) and context (health services and systems) [22]. Data were managed using EndNote X20 software.

### Data charting and synthesis

A data extraction sheet was developed covering author, year, country, types of study, main concepts, and key findings related to the research question (Appendix, Table S2). We used Braun and Clarke's inductive thematic analysis approach [23]. Themes were explained narratively using a multilevel (individual, organizational, and system levels) framework [24]. Individual-level care coordination/continuity of care generally refers to relational continuity of care (provider and patient relation in the uptake of services at different times and settings), care coordination at organizational level focuses on informational continuity (communication among professionals for multidisciplinary care), while system-level care coordination refers to management continuity (coordination of systems and services) [6].

## Results

**Fig. 1:**
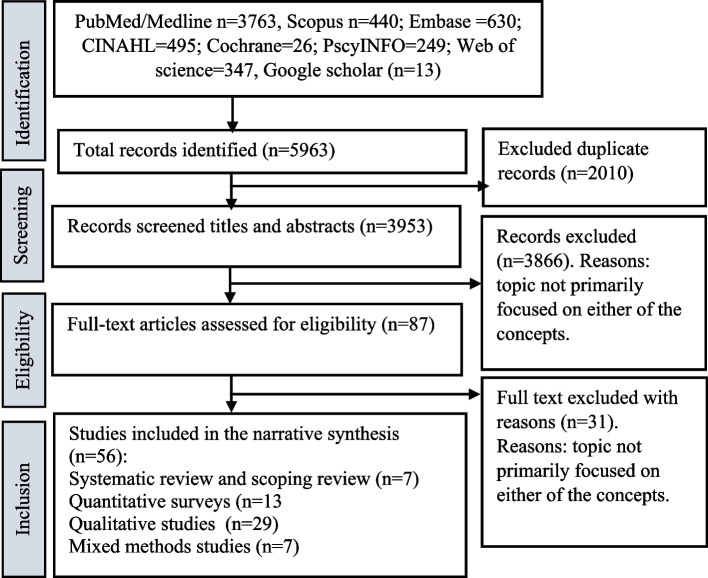
PRISMA flowchart showing selection of studies included in the review

A search of all databases yielded 5963 articles (Fig. 1). We removed 2010 duplicated studies, and 3953 articles were screened for relevance based on title and abstract, whereas 3866 articles were excluded, leaving 87 articles for full-text screening. In addition, 31 articles were excluded after the full-text screening with reasons. Finally, 56 studies were included in the review.

### Distribution of studies

Of a total of 56 studies, there were 32 studies from high-income countries (HICs), 11 studies from upper-middle-income countries (UMICs), nine studies had unspecified geographical locations and four studies from low and lower-middle-income countries (LLMICs) (Table 1). Studies from HICs included the USA (7), five each from Australia, and the Netherlands, Canada (3), two from South Korea and the UK, and one each from 7 countries (European Union, Finland, Germany, Norway, Taiwan, Sweden, Northern Ireland). Studies from UMICs included Brazil (9), Indonesia (2), and one each from Argentina, China, Malaysia, and South Africa, and four LLMICs were Nepal, Nigeria, Pakistan, and Tajikistan. Additionally, Table 1 presents themes and numbers of studies under a multilevel framework: individual (blue shaded), organizational (yellow) and system (green) levels.

**Table 1:**
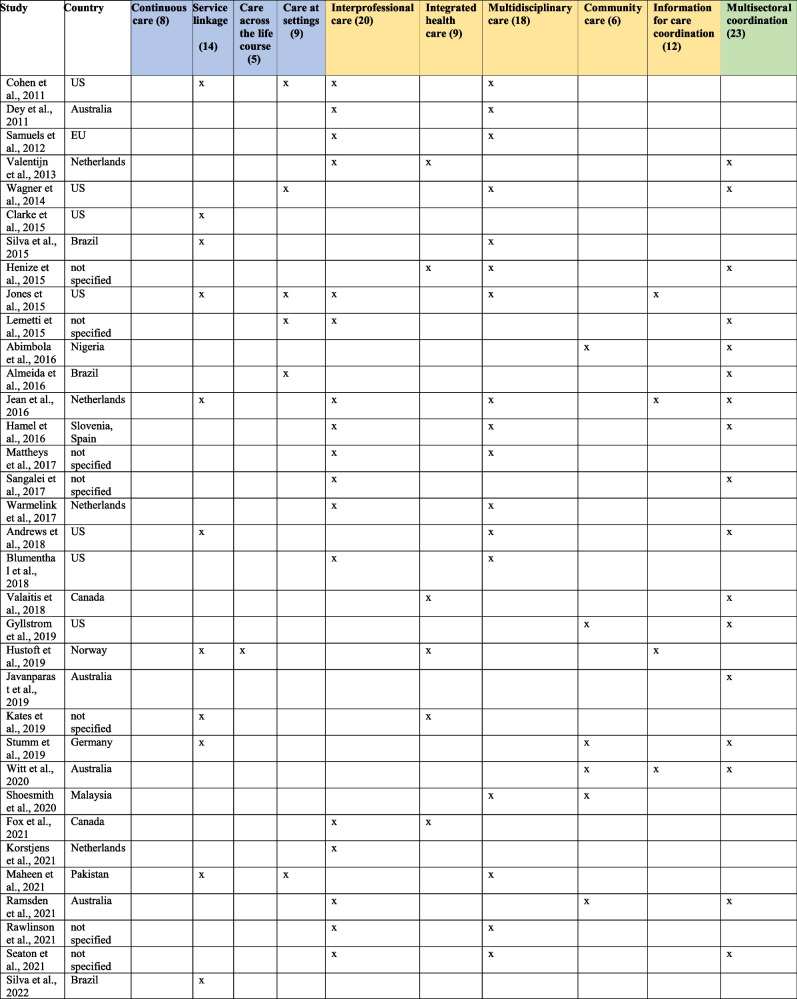

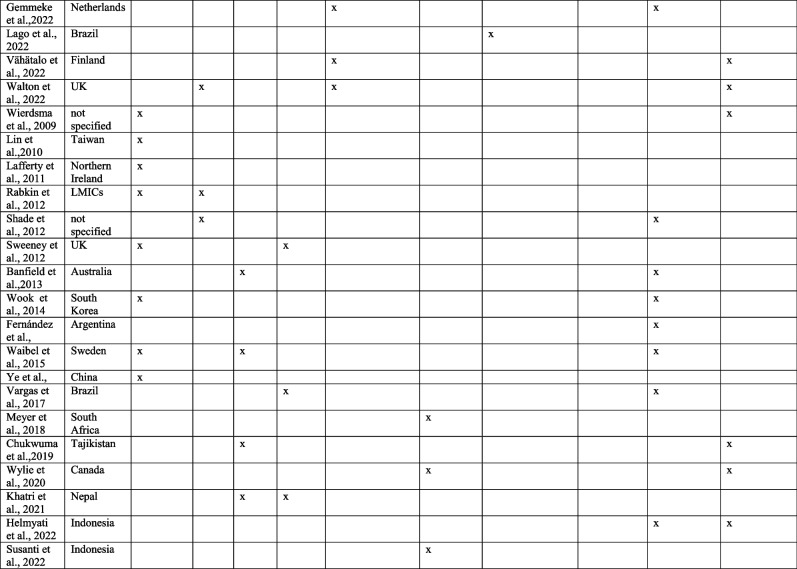
Care coordination and continuity of care mapping at individual, organisational and system levels [25–80]

### Individual-level care coordination

Four themes at this level included: continuous care, service linkage, care across life-course and care coordination at place dimensions.

#### Continuous care

Eight studies reported continuous care of PHC or primary care. Continuity of care is continuous over time; it involves the relationship between the health workers and patients built on trust, loyalty, and constancy of an individual patient. Care coordination is relational and contact continuity, and cross-boundary care is recorded objectively [63, 65]. Patients in the high continuity of care group underpinned by preconditions, staff-related continuity, and care contacts presented more remarkable improvement in the functional role of physical, general health, emotional, and mental health than the low continuity of care [68, 73]. Chronic care systems, lessons and resources were leveraged to support people with HIV-negative with chronic NCDs [66]. Care of chronic diseases (chronic obstructive pulmonary diseases and diabetes) found continuity of clinical management, such as distribution of care across levels and rapid access and referral to reduce future hospitalisations and long-term diabetic complication admissions [64, 72]. Contrarily, low continuity of care was also associated with increased inpatient and outpatient days and costs in cardiovascular diseases [70]. Poor continuity of care increased the chance of hospital admission [64].

#### Service linkage from prevention to rehabilitation

Fourteen studies included linkage of services in PHC and primary care services. The linkage of a wide range of services improved continuity of care. For instance, a range of services was needed according to disease progression with emphasis on coordinated care efforts (prevention, screening, treatment, and rehabilitation services) [33, 46]. Such service linkages were found effective in reducing clinical burden, reinforcing the role of the care providers in delivering care prevention, detection, and treatment of diseases [48]. Nonetheless, studies suggested high discontinuation of health service coverage in the care continuum (health promotion, access to services, real-time case finding, treatment), influencing service optimisation and closing gaps [42, 67].

Service linkage, along with disease progression, was pivotal to providing health services among people with comorbidities. Patients with multiple chronic conditions require an assessment, comprehensive care, self-management support, linkage of community resources, monitoring, and follow-up [30, 49]. For example, older people living with HIV required treatment for multiple diseases as they were a risk for rapid progression of disease and complications of NCDs (e.g., hypertension, diabetes, and cancers) [66]. Delegation of responsibilities from general practitioners to qualified health staff and practice teams with a wide range of tasks and their deployment was found to support coordination tasks, and social and legal duties [30, 49].

Provider-related successful strategies of linking services in care coordination included increased frequency of appointments (regular, on demand, hybrid), access to records (full or filtered access), modes of care (face-to-face, digital, telephone), counselling (telephone, web-based), and practices of care [30, 62]. Additionally, self-management/self-care by service users enhanced care coordination of a range of services [25, 37].

Challenges of service linkages in the coordination of care were fragmented care, lack of provider cooperation, inadequate awareness/health literacy on health issues and care, lack of access, recording of complaints, scheduling of return visits including written referrals, and clarifying information about referral services [54, 58]. The linkage of preventive and treatment services in multimorbidity was influenced by the roles of providers, patient health behaviour, and cooperation within the complex care system [25, 31, 49].

#### Care across the life-course

Five studies reported care coordination across the life-course perspectives. Healthcare coordination across life-course involves healthcare needs and delivery from conception to death. Health conditions and disease progression do not go in a linear pathway along the life course. Instead, health services require according to health conditions across the life course. Measures of patient experiences with different types of care provided continuity of care and had a promising indicator of the quality of care to change in patient-rated health [46]. In Nepal, there was low completion of (41%) of maternity care in the antenatal to postnatal period, with high discontinuation around childbirth and among women from disadvantaged ethnicities, low wealth status, illiterate, and remote areas [78]. Influencing factors of poor care coordination included insufficient availability of information, long waiting times, unclear roles, inadequate referrals, and staff turnover [69, 72]. In hypertension treatment, discontinuation in a cascade of care was influenced by misinformation, ambiguous protocols, and limited delivery capacity [76].

#### Care coordination across place dimension (from home to hospitals)

Nine studies included care coordination of PHC and primary care services in place dimension. Healthcare delivery depends on the settings starting from home to hospitals. For instance, promotive and preventive interventions can be delivered to the family and community. While first level of care (often referred to as primary care) is available at peripheral health facilities (e.g., health posts), and secondary and tertiary care are available in hospitals. Care coordination was positively correlated with patient-centred medical home assessment items such as referral or transition process, connections to support information exchange, and consistency of doctors and health needs [29, 74]. In addition, point-of-delivery reminders and decision support have facilitated the coordination of health behaviour counselling for primary care patients [25].

In Brazil, PHC (as the first contact of preference) faced strong competition from hospital outpatient and emergency services outside the network [36]. The collaboration of nurses in hospitals and PHC settings was an integral part of nurses' work; hospital-admitted patients experienced high continuity of care [34, 68].

In a hospital, challenges were difficulties in obtaining health services, timely follow-up appointments for after-hours or weekend discharges, lack of awareness of hospitalization, not having hospital records for post-hospitalization appointments, difficulty locating information in discharge summaries, feeling undervalued when hospitalists made medication changes without involving providers [33]. Factors influencing the discontinuity of maternity care and quality of life included the deficient counter-referral system, communication, lack of respect, and lack of empowerment [36, 54, 68, 79].

### Organization-level care coordination

Five themes under this level included interprofessional care, integrated health care, multidisciplinary care, community care and information for care coordination. At this level, care coordination refers to the delivery of services through providers and the community.

#### Interprofessional care

Twenty studies reported interprofessional care of PHC and primary care services. Experience teamwork and interprofessional collaboration of HCPs were vital for delivering PHC services [28]. Organizational factors for care by interprofessional teams included attributes of evaluating the level of collaboration within teams and networks for good practices [27]. Additionally, coordination of interprofessional practices among providers (e.g., coordination among pharmacists, general practitioners, and nurses) improved teamwork in delivering health services [39, 40, 52]. For example, in Australia, team dynamics of general practitioners and pharmacists articulated professional relationships in primary care highlighted a pathway to more collaborative practices [26]. Furthermore, multidisciplinary team agreements on providers' roles and responsibilities were stimulated in fall prevention programs [59].

Interprofessional collaborative care ensured coordinated care addressing predictable issues, making patients safer in hospitals for better outcomes [34, 43]. Allied health professionals working near health practitioners from other professions (physicians and nurses) regularly interacted with hospitalized patients for improved satisfaction and outcomes [39, 57]. Interprofessional practices in PHC emphasized the benefits of occupational health services by developing tools or guidelines for successful implementation [55, 56, 61]. In the Netherlands, maternity care providers such as midwives' interactions with physicians and their interprofessional relations enhanced primary care in urban areas [41]. Opportunities for frequent communication and relationships, clinical interaction, recognition of other professionals' skills, training, and referral were essential for interprofessional communication [25, 33, 56, 57]. Strategies of multidisciplinary care coordination included organizing care from model (local, hybrid, national) or involvement of care (collaboration between many or all individuals, collaboration between some individuals), interpersonal factors (e.g., language differences, knowing each other, trust, and respect), professional-related factors (e.g., individual competencies, motivation), internal (e.g., structure, composition) and shared vision [37, 62].

In Spain, a collaboration of nurses and general practitioners and incentives are encouraged towards improving teamwork [38]. In addition, informal communication among providers and maternity care users (talk and work, small talk and humour, work beyond words, familiarity, use of sight, touch, sound, and non-verbal gestures) was found effective in providing and delivering health services [53].

Poor knowledge about the availability of services, time and training, lack of clear roles, fears relating to professional identity and poor communication, poor understanding of contexts challenges appearing to lead to weak mutual trust, lack of cooperation, a poor collaboration including lack of agreements with pharmacists, limited coordination and communication [56, 59, 61]. Limited partnerships with community pharmacists and allied professionals in fall injury prevention influenced limited knowledge of drugs, and the potential role of pharmacists [59]. Additionally, in some instances, conventional power structures between professions hinder teamwork and interprofessional collaboration between care providers [38, 40].

#### Integrated health care

Nine studies reported care coordination as integrated health care. In public health and primary care, access to person-focused and population-based care collaboration had complementary roles in clinical integration, coordination, and patient connections with community organizations [28, 77]. Dimensions of integrated care that enhanced patient-rated care continuity for complex problems included a better understanding of the complex inter-relationships and interactions of public health functions in primary care, communication, and relationships [28, 44, 46, 48]. Drivers of collaborative and integrated primary care included communications, understanding together, risk assessment, follow-up and tracking organizing and prioritizing risks and interventions, and operationalization in clinical settings [32, 52, 75].

The use of mHealth (risk factors and treatment) was effective integrated care, such as in developing competency indicators and purposes and benefits within the scope of continuity of care [80]. An integrated Community Oriented Primary Care approach increased pregnancy care at home; however, the challenges of mobilizing the ward-based outreach team were lack of patient residence or personal identification [75].

#### Multidisciplinary services

A total 18 studies described care coordination through provision of multidisciplinary services. Care coordination of multiple services as delivered from multidisciplinary primary care, termed the co-locations within the same physical space and offered opportunities for interprofessional collaboration [25, 33, 56, 57]. Skills mix and task shifting through interdisciplinary collaboration enhanced healthcare providers' education, workforce adaptation, and occupational structure and skill mix in primary care [25, 27, 37].

Multidisciplinary workforces fill the gaps in interdisciplinary services at the service delivery point. Evidence revealed care coordination by ensuring multidisciplinary services; for instance, in Pakistan, the existing primary health structure provided a foundation to deliver multiple benefits and maintain continuity of care [54], while in Slovenia, a collaboration between general practitioners and nurses, their organization in separate care units (case-oriented functions) offered integrated advanced practice nurses into general practice, shared vision of preventive care and strengthened attitudes towards team-oriented care [38]. Collaboration actions of physicians and nurses positively contributed to patient outcomes midwives with more work experience were satisfied with their collaboration with general practitioners [39, 41]. The collaboration of paediatricians and community partners leveraged their strengths and shared vision to improve healthy families and the well-being of at-risk children [32]. Interactions and team-based care of non-physician care providers (e.g., midwives) in established health centres had higher satisfaction in collaboration in the care continuum, encompassing nurses’ autonomous role in the care process and reducing professional disagreements [38, 41].

Implementing core functions of care coordination included easy-to-use system-level solutions, automated prompts and decision support tools, training of health workforce, integration of electronic health records, data and case reviews, quality improvement methods, and innovative use of clinical space were essential components in different clinical settings [25, 32, 37, 42]. In addition, elements of care coordination for patient safety in primary care settings included supporting patients (e.g., referral or transition process), creating connections to support information exchange, a culture of accountability and team dynamics, precursors (opportunity to participate, knowledge and shared objectives), elements (competency, awareness and understanding of work roles and interaction), and outcomes (events or behaviours as consequences of collaboration of providers at facilities) [29, 33, 34, 43].

Barriers to multidisciplinary care included lack of time, difficulty reaching other clinicians, lack of personal relationships with other clinicians, lack of information and feedback loops, discrepancies in medication list, lack of clarity on accountability and autonomy, relatedness, motivation and resources, and the potential cost of care without reciprocation [33, 51]. Furthermore, mismatch and relationships of providers hindered multidisciplinary care coordination (e.g., general practitioners and pharmacists, professional groups), disputes, physician-centred power, damage of shared care, resistance to interprofessional collaboration based on knowledge-power relations, and lack of knowledge on interprofessional interference [26, 60]. For instance, the effects and results of fragile coordination in Children's PHC in Brazil were divergences of health units in the organization of care, delays obstructing access to technologies, and absence of effective communication and lack of medical transport [31].

#### Community care continuity

Six studies explained community care continuity of PHC and primary care services. Several community factors can influence care coordination in PHC services. Community Collaboration Health Model with wide variation in relationship factors (e.g., foundational characteristics) promoted sustainability or innovation [45]. In Australia, collaborative approaches of Indigenous community organizations streamlined flexible care delivery, patient-centred care and support processes, timely communication, and information exchange [50]. Such community engagement approaches enhanced building health workforce literacy, town-based planning for improvement for the continuity, care coordination primary care and hospitals services (PHC service and treatment) [50, 55]. In Nigeria, the health facility committee’s decision involved coordination to co-produce formal health services, facilitation of referrals from informal to formal providers, and reduction of the market share controlled by regulating informal providers, making competitive formal providers, leveraging the authority and resources available within their community [35].

In Malaysia, community factors of continuity of care include collaborative behaviours, motivation towards a common goal or value, autonomy, relatedness (e.g., trusting, understanding and caring about the other), resources (e.g., competence, time, physical resources and opportunities), and motivation for collaboration (weighing up the personal costs versus benefits of acting collaboratively) patients [51]. Nonetheless, inefficient communication with healthcare providers, a slow and faltering process of institutional change with a make-or-buy decision, and efforts barred patient access to care and outcomes [35, 49, 51].

#### Informational continuity

There were 12 studies that discussed information continuity of care to deliver PHC services. Effective communication channels and a formal structure for functions support decision-making were strategies for redesigning the contingency plan and strategic actions. Shared information reduced unnecessary repetition and allowed HCPs to access records of care [69]. The electronic monitoring and evaluation system implemented and completed several modifications to accommodate and increase users’ engagement in personal care rather than the passive availability of information [67, 79]. Accessibility and continuity of health information exchange interventions to access various data systems and patient information (e.g., surveillance, electronic health records, laboratory, and billing) focused on improving linkage and retention, quality, and efficiency of care [67, 69]. Direct access to communication technology and incorporation of technological contingencies in supervision and redefining clinical information improved the exchange of information, shared electronic medical records, social sensitivity and self-reflection, and referral criteria towards in primary care and patient care [33, 37, 46, 53, 59, 71]. Chronic diseases such as cancer treatment require continuity of information (transferred across levels via communication mechanisms) and continuity of relationships (patients of networks), especially in marginalised populations [50, 72].

Nonetheless, there were low levels of informational continuity insufficient for complex conditions, including transfer and care coherence influencing ongoing patient-doctor relationships [69, 74]. In addition, data ownership and confidentiality also hampered information sharing and associated responsiveness [69].

### System-level care coordination

Under system-level theme was: **multisectoral care coordination within and beyond health systems**. Of 56 studies, multisectoral care coordination was described in 23 studies. At this level, care coordination refers to organizations of systems and services incorporating coordination within technical departments of organisations (deals for health services) and supportive departments (e.g., finance, administration, logistics) and employing multisector and stakeholders within and beyond organisations and systems.

System-level care coordination is essential for health service management (ensuring inputs and facilitating processes) towards the delivery of services. Multisectoral coordination for health service management worked based on a shared philosophy, financial considerations, leadership strategies, power, and hierarchy [42, 57]. Multiple sectors were involved at the management level for collaborative health policy/planning and funding, community engagement, private sector engagement (to provide specialized care), building health workforce, and system changes across services and settings [36, 50, 55, 63].

Higher level system integration (governments, NGOs, donors, and international agencies) was dedicated to reimbursement redesign, improvement of data systems and sharing capability towards cooperation resources [28, 45, 61]. Furthermore, at the community level, the health committee enhanced the sectoral coordination for authority and resources from governments and supporting agencies [35]. Furthermore, collaborative efforts within the health system for care coordination included planning for systemic and healthcare training needs, creating a resilient health system for anticipating and adapting to adverse situations, scaling up screening through health caravans, task-shifting, and introducing job aids for providers [76, 77, 79]. Additionally, coordination and collaboration with other sectors in health enhanced shared vision, advocating for public health policy, mobilizing faith-based organizations, codeveloped plans for implementation and evaluation, resource alignment, structures, and financial incentives [32, 38]. Leadership, administrative support, responsibility for coordination, accountability, building relationships with care partners, and external factors (e.g., culture, hierarchy, regulations, finance, and communication technology) [29, 34, 37, 62].

Multisectoral collaboration improved resources for organizational capacity, collaborative planning, supportive governance, and public health legislation to tackle social determinants of health by mandating the role of local governments and institutionalizing PHC services [36, 47]. Implementing and maintaining collaboration between partners, and organizational leaders needed to identify shared priorities, joint reflection and adaptation, shared decisions, achieving tangible benefits, realisation of sustained relationships, clearly defined structures, shared visions of care, team development, optimal use of resources, and collaborative approaches to programs and services [32, 38, 44, 45].

System-level challenges (administrative, organizational, structural, and relational), the lack of standard measures and administrative data hindered the capacity of health providers to ensure continuity of care [40, 49, 63]. For example, in Brazil, essential attributes influencing care coordination were fragmentation of the regional network, inherent problems in PHC, and poor coordination capacity [36]. In Australia, Medicare Locals and Primary Health Networks reported limited time and financial support for cooperation with the local government due to the inadequate collaborative capacity of local organizations in tackling issues [47].

## Discussion

This scoping review identified health care coordination at the individual, organizational and system levels. Care coordination in health services is vital for continuous care at different stages of health conditions across life courses and settings for delivering a wide range of health services. Organizational care coordination included interprofessional, multidisciplinary services, community continuity of care, information for care, and integrated health care (public health and primary care). Care coordination at the system level involves the coordination of multiple sectors within and beyond for synergistic efforts in organizing and generating health services.

Care coordination at the individual level focuses on individuals with specific health needs across the life course and places which requires coordination of different levels of services (e.g., preventive to rehabilitative care). The care coordination model at the individual level can enable the safety net of primary care/PHC for individuals by building relationships with care providers, supporting patients, and facilitating the development and evaluation of existing and new care models for health needs, settings and life-course perspectives [29, 62]. For example, promotive interventions (such as lifestyle modifications and behaviour change) could promote people's health and well-being. In contrast, interventions related to screening can identify high-risk populations and early diagnosis of illnesses [7]. The health coordination links various health services from prevention to treatment of disease conditions [30]. Detection, treatment, and rehabilitation are approaching the disease progression and needs, which can be delivered in different settings [42]. For example, preventive services can be delivered at the home while screening services at the community level, and screening and treatment services can be received at the facility settings [12, 54, 81]. Coordination of care across settings permits integrating services to address the patient's and family's comprehensive needs that can result in efficiency (e.g., decreased healthcare costs, fragmented care, and improvement in care practices) [82].

Organizational care coordination involves multidisciplinary providers and services, vital for supplying integrated care (public health and primary health care). Professional training and cultural competency are essential for new or existing service collaborations [27, 44, 53]. The PHC workforce can improve access to and quality health care services [55]. Developing a care pathway across the hospitals led to using existing and newly constructed structures and data monitoring [83].

Current health systems and model of care focuses on primary care on treating illnesses but less focus on health prometon and prevention. Such disease-focused models cannot meet individual and community health needs; instead, they require people-centred integrated health services to address changing epidemiological and demographic shifts. For instance, children and youth with special health care needs require interfacing multiple care systems (home care agencies, advocacy groups, allied health services) and professionals (medical, social, and behavioural), and families [82].

Multidisciplinary services provided through interprofessional collaboration could meet the changing burden of diseases and emerging health needs of the population by integrating public health interventions focusing on health and conditions [84]. Additionally, technological advances can be driving forces for transforming primary care to reach disadvantaged populations [84]. For example, family health (interdisciplinary team) can provide various care options for health service delivery in public health emergencies by ensuring infection control, coordination, antiviral medication, clinical care, nursing services, and communication strategies [85]. In addition, the community health workers approach can bring patient-centred infrastructure and resourcing, unique liaison capacity for people, communities, and health facilities, and evolving care continuum across patient ages to promote health equity and reduce the cost of care [86].

Furthermore, information in the continuity of care is best suited to manage the relational continuity among multidisciplinary providers and integrated care. Therefore, organisational efforts are needed to consider the broader fit for the provider's local responsiveness [69].

Within the health system, it is vital to ensure coordination of departments/units in the organizations to provide and deliver health services. For example, organizations' finance, administration, and logistic teams play essential roles in the organization of services. These units play a supporting role in providing and delivering health services and facilitate the health care coordination for the organisations, providers, and service users. Additionally, the role of multiple stakeholders has an indirect impact on health services. Coordinating multiple sectors (health and non-health) and stakeholders (governments, non-government, private sector) are the backbones of resource mobilization, health planning and health system governance. A broader system-level collaboration is required to mobilise resources for better health planning and management of health services [47, 48, 55]. Interorganizational care coordination suggests that partners’ efforts to align their coordination domains can improve health care delivery [87].

This study provided some insights into care coordination at different levels. Firstly, care coordination conceptualizes health services and providers at individual, organisation and system levels and intersects at three levels. Secondly, the delivery of health services requires according to the health conditions; for instance, the early stage of disease progression requires health promotion, followed by prevention, screening, treatment and rehabilitation. Thirdly, implementing interpersonal or multidisciplinary collaboration requires relational and informational continuity among the providers and services within the health facilities. Fourthly, management continuity is more incredible within and beyond the health system to ensure continuous provision and delivery of health services [69]. Finally, care coordination is widely accepted and discussed in HICs primarily focusing on hospital primary care and care. However, it is imperative that LMICs also require to focus on research on health care coordination to identify strategies and approaches and inform policy and practices.

This study has some limitations. We have not conducted quality appraisal of studies and included studies written only in English. Our purpose of the review was to synthesize evidence rather than grade the evidence. Synthesized evidence from this study could provide research, policy, and program insights for the improved care coordination of PHC/ primary care. In addition, we conducted a thematic synthesis of evidence on the care coordination services to answer the research question. However, such analysis can miss country-specific findings and specific subsections of populations. Future research can be conducted on specific components of care coordination (such as life course perspective) or care coordination at a specific level (for instance, care coordination at the organisation level for delivery of health services) focusing on specific populations, health conditions, or health interventions.

## Conclusions

Care coordination involves health care providers in the course of disease progression, life course perspectives and places of care (family to health facilities). The care coordination/continuity of care conceptualises from the lens of utilization of services, health conditions-specific services to individuals, delivery of health services collaborating with multiple professionals or multiple services, and management of health services with support from other non-health departments, stakeholders within health systems and beyond. Several issues influence the care coordination at the individual (services or users), organizational (health care settings where ranges of providers are working) and system levels (where organisations and sectors involve in care coordination). Health system efforts focus on care coordination, ensuring types of care per the healthcare needs at different stages of health conditions by multidisciplinary professionals and coordinating multiple technical and supporting stakeholders and sectors within the health systems. In addition, health system efforts require more emphasis on ensuring care coordination in research and practices in LMICs, especially in PHC system settings.

## Supplementary Information

**Additional file 1:** **Supplementary information, Table S1.** Preferred Reporting Items for Systematic reviews and Meta-Analyses extension for Scoping Reviews (PRISMA-ScR) Checklist. **Supplementary information, Table S2.** Data extracts of the findings of coordination in PHC.

## Data Availability

All data generated or analyzed during this study are included in this published article [and its supplementary information files].
