# Supplementary material for: Continuity and care coordination of primary health care: a scoping review
Source: BMC Health Serv Res. 2023 Jul 13;23:750. doi: 10.1186/s12913-023-09718-8 (PMC10339603; doi:10.1186/s12913-023-09718-8)
Supplement: Supplementary file 1 — Additional file 1: Supplementary information, Table S1. Preferred Reporting Items for Systematic reviews and Meta-Analyses extension for Scoping Reviews (PRISMA-ScR) Checklist. Supplementary information, Table S2. Data extracts of the findings of coordination in PHC. [file 12913_2023_9718_MOESM1_ESM.docx]

# Supplementary file

**Supplementary information, Table S1: Preferred Reporting Items for Systematic reviews and Meta-Analyses extension for Scoping Reviews (PRISMA-ScR) Checklist**

| SECTION | ITEM | PRISMA-ScR CHECKLIST ITEM | PAGE # |
| --- | --- | --- | --- |
| TITLE | | | |
| Title | 1 | Identify the report as a scoping review. | 1 |
| ABSTRACT | | | |
| Structured summary | 2 | Provide a structured summary that includes (as applicable): background, objectives, eligibility criteria, sources of evidence, charting methods, results, and conclusions that relate to the review questions and objectives. | 2 |
| INTRODUCTION | | | |
| Rationale | 3 | Describe the rationale for the review in the context of what is already known. Explain why the review questions/objectives lend themselves to a scoping review approach. | 4 |
| Objectives | 4 | Provide an explicit statement of the questions and objectives being addressed with reference to their key elements (e.g., population or participants, concepts, and context) or other relevant key elements used to conceptualize the review questions and/or objectives. | 4 |
| METHODS | | | |
| Protocol and registration | 5 | Indicate whether a review protocol exists; state if and where it can be accessed (e.g., a Web address); and if available, provide registration information, including the registration number. | NA |
| Eligibility criteria | 6 | Specify characteristics of the sources of evidence used as eligibility criteria (e.g., years considered, language, and publication status), and provide a rationale. | 5 |
| Information sources* | 7 | Describe all information sources in the search (e.g., databases with dates of coverage and contact with authors to identify additional sources), as well as the date the most recent search was executed. | 4-5 |
| Search | 8 | Present the full electronic search strategy for at least 1 database, including any limits used, such that it could be repeated. | 5 |
| Selection of sources of evidence† | 9 | State the process for selecting sources of evidence (i.e., screening and eligibility) included in the scoping review. | 5 |
| Data charting process‡ | 10 | Describe the methods of charting data from the included sources of evidence (e.g., calibrated forms or forms that have been tested by the team before their use, and whether data charting was done independently or in duplicate) and any processes for obtaining and confirming data from investigators. | 6 |
| Data items | 11 | List and define all variables for which data were sought and any assumptions and simplifications made. | 6 |
| Critical appraisal of individual sources of evidence§ | 12 | If done, provide a rationale for conducting a critical appraisal of included sources of evidence; describe the methods used and how this information was used in any data synthesis (if appropriate). | 4 |
| Synthesis of results | 13 | Describe the methods of handling and summarizing the data that were charted. | 4-6 |
| RESULTS | | | |
| Selection of sources of evidence | 14 | Give numbers of sources of evidence screened, assessed for eligibility, and included in the review, with reasons for exclusions at each stage, ideally using a flow diagram. | 7 |
| Characteristics of sources of evidence | 15 | For each source of evidence, present characteristics for which data were charted and provide the citations. | 8 |
| Critical appraisal within sources of evidence | 16 | If done, present data on critical appraisal of included sources of evidence (see item 12). | Page 5, and 23 |
| Results of individual sources of evidence | 17 | For each included source of evidence, present the relevant data that were charted that relate to the review questions and objectives. | 8-19 |
| Synthesis of results | 18 | Summarize and/or present the charting results as they relate to the review questions and objectives. | 8-19 |
| DISCUSSION | | | |
| Summary of evidence | 19 | Summarize the main results (including an overview of concepts, themes, and types of evidence available), link to the review questions and objectives, and consider the relevance to key groups. | 19 |
| Limitations | 20 | Discuss the limitations of the scoping review process. | 23 |
| Conclusions | 21 | Provide a general interpretation of the results with respect to the review questions and objectives, as well as potential implications and/or next steps. | 23-24 |
| FUNDING | | | |
| Funding | 22 | Describe sources of funding for the included sources of evidence, as well as sources of funding for the scoping review. Describe the role of the funders of the scoping review. | 24 |

# Supplementary information, Table S2: Data extracts of the findings of coordination in PHC

| **Study** | **Type** | **Country** | **Aim** | **Key Themes** | **Key Findings** |
| --- | --- | --- | --- | --- | --- |
| Cohen et al., 2011[25] | Qualitative | US | To examine how coordinated care is implemented in primary care practices to address patients' health behavior change needs. | Coordinated care practice for patient behaviour.  Easy-to-use system-level solutions | Automated prompts and decision support tools, brief counselling and referral, training, and co-location of referral with outreach implementation. Reduce the clinical burden through telephone and Web-based counselling systems or by expanding the medical assistant role in coordination of health behavior counselling experienced difficulties in implementation. Have point-of-delivery reminders and decision support facilitated coordination of health behavior counselling for primary care patients. |
| Dey et al., 2011 [26] | Qualitative | Australia | To gain understanding of expectations, experiences and perceptions of GPs and pharmacists. | Minimal collaboration of GPs and pharmacists. | Minimal collaboration, mismatch and relationship between GPs and pharmacists in patient care among professional groups. A model for the development of GP-pharmacist relationship can articulate the dynamic nature of professional relationship in primary care and highlights a pathway to more collaborative practice. |
| Samuels et al., 2012 [27] | Qualitative | European Union | To analyse how to improve Interprofessional collaboration (IpC) within primary care teams | Factors and condition of interprofessional collaboration | Conditions to improve IpC included education of healthcare professionals, adapting human resources, the occupational structure, and the skill-mix in primary care. Several contextual factors influence the development of IpC at different levels. A framework is required to evaluate the level of collaboration within teams and network for good practice. |
| Valentijn et al., 2013 [28] | Qualitative | Netherlands | To understand the concepts of primary care and integrated care, complexity of integrated care. | Integration of primary care.  Multilevel integration | Guiding principles for achieving integration are person-focused and population-based care at the three levels such as integration plays complementary roles on the micro (clinical integration), meso (professional and organisational integration) and macro (system integration) level. Functional and normative integration ensure connectivity between the levels to achieve a better understanding of the inter-relationships among the dimensions of integrated care from a primary care perspective. |
| Wagner et al., 2014 [29] | Quantitative | US | To examine the utility of a newly developed Care Coordination Model in improving care coordination among participating practices | Elements of Care Coordination Model | Positively correlated with some PCMH-A care coordination items were accountability, build relationships with care partners, support patients through the referral or transition process, and create connections to support information exchange. Activities consistent with the 4 elements of the Care Coordination Model can enable safety net primary care to better coordinate care for its patients |
| Clarke et al., 2015 [30] | Quantitative | US | To implement and evaluate a program that embedded non licensed coordinators in primary care practices | Coordination of care | Comprehensive care coordinators (CCC) interventions included execution of care, coordination of transitions, self-management support/link to community resources, monitor and follow-up, and patient assessment. The CCC intervention group had a 20% greater reduction in its pre-post emergency department (ED) visit, and reduction in ED visits by focusing on the centrality of the primary care provider and practice. |
| Silva et al., 2015 [31] | Qualitative | Brazil | To identify the attribute ‘coordination’ in primary healthcare, in the perception of parents/caregivers, for resolution of health problems in infants. | Coordination of care among infants | Effects and results of fragile coordination in Children's PHC included: Divergences between different health units in the organization of care; functional barriers and delays obstructing access to technologies; absence of effective communication; absence of medical transport; need for healthcare on an individual basis; and coordination involving management of healthcare. Absence of coordination results lack of a solution-based approach in healthcare for infant, gaps in the organization of the services and in health management. Communication, access to technologies, referral and counter-referral systems, and secure transport are essential for organizing primary care services and offering rounded care to child. |
| Henize et al., 2015 [32] | Qualitative | not specified | To offer a roadmap to help structure primary care approaches to these needs through the development of comprehensive and effective collaborations between the primary care setting and community partners. | Collaborations with community organizations- shared vision, codeveloped plans for implementation and evaluation, resource alignment | Phase collaborative approaches in primary care: build the case for action through a family-centered risk assessment, organize and prioritize risks and interventions, develop, and sustain interventions, and operationalize interventions in the clinical setting. Phased approach of collaboration includes shared vision, codeveloped plans for implementation and evaluation, resource alignment, joint reflection and adaptation, and shared decisions regarding next steps. Training, electronic health record integration, refinement by using quality improvement methods, and innovative use of clinical space are important components that may be useful in a variety of clinical settings. Paediatricians and community partners can collaborate to improve the well-being of at-risk children by leveraging their respective strengths and shared vision for healthy families. |
| Jones et al., 2015 [33] | Qualitative | US | To understand the challenges in coordination of care, as well as potential solutions, from the perspective of hospitalists and PCPs. | Successes and challenges of care coordination | Care coordination challenges included lack of time, difficulty reaching other clinicians, lack of personal relationships with other clinicians, lack of information feedback loops, medication list discrepancies, and lack of clarity regarding accountability for pending tests and home health. Hospitalists noted difficulty obtaining timely follow-up appointments for after-hours or weekend discharges due to lack of awareness on hospitalisation, not having hospital records for post-hospitalization appointments, difficulty locating important information in discharge summaries, and feeling undervalued when hospitalists made medication changes without involving PCPs. Themes of successful care coordination included greater efforts to coordinate care for “high-risk” patients, improved direct telephone access to each other, improved information exchange through shared electronic medical records, enhanced interpersonal relationships, and clearly defined accountability. Challenges in care coordination included sending and receiving roles for hospital discharges. |
| Lemetti et al., 2015 [34] | Review | not specified | To understand collaboration between nurses working with adults in hospital and primary health care, and to facilitate the future measurement of this collaboration. | Process of collaboration | Process of collaboration included collaboration precursors (opportunity to participate, knowledge and shared objectives), elements of collaboration (competency, awareness and understanding of work roles and interaction) and processes and outcomes (the events or behaviours that are the consequences of the collaboration between hospital and primary healthcare nurses). Collaboration between hospital and primary healthcare nurses is integral part of the work of nurses and a process consisting of several predictable issues leading to useful care outcomes. |
| Abimbola et al., 2016 [35] | Qualitative | Nigeria | To explore the role that communities could play in limiting informal providers | Community health committees and coordination mechanism | The committees influence in a slow and faltering process of institutional change, leveraging the authority and resources available within their community, and from governments and NGOs. Committee provide information to reduce the market share controlled by informal providers, then regulation to keep informal providers at bay while making the formal provider more competitive. Committees are faced with a “make-or-buy” decision and efforts are inefficient and insufficient and made decision involves coordination to co-produce formal health services and facilitate referrals from informal to formal providers. |
| Almeida et al., 2016 [36] | Quantitative | Brazil | To analyse the breadth of care coordination by PHC in three health regions. | Care coordination in specialized care and primary care | PHC as first contact of preference faced strong competition from hospital outpatient and emergency services outside the network. Issues related to access to, and provision of specialized care were aggravated by dependence on the private sector in the regions, despite progress observed in institutionalizing flows starting out from PHC. The counter-referral system was deficient and interprofessional communication was scarce, especially concerning services provided by the contracted network. Coordination capacity is affected both by the fragmentation of the regional network and intrinsic problems in PHC, which poorly supported in its essential attributes. |
| Jean et al., 2016 [37] | Qualitative | the Netherlands | To explore influential factors regarding interprofessional collaboration related to care plan development in primary care. | Factors influencing the interprofessional collaboration | Factors of interprofessional collaboration included patient-related factors ( active role, self-management, goals and wishes, membership of the team), professional-related factors (individual competences, domain thinking, motivation; interpersonal factors: language differences, knowing each other, trust and respect, and motivation), organisational factors (structure, composition, time, shared vision, leadership and administrative support), and external factors ( education, culture, hierarchy, domain thinking, law and regulations, finance, technology and ICT). |
| Hamel et al., 2016 [38] | Literature review | Slovenia and Spain | To comparative analysis of concepts and practices of GP-nurse collaborations in primary health centres in Slovenia and Spain. | Collaboration between GPs and nurses. Conventional power structures between professions hinder effective collaboration. | In Slovenia, the collaboration between GPs and nurses has been strongly shaped by their organisation in separate care units (case-oriented functions), integrated advanced practice nurses into general practice, shared vision of preventive care is gradually strengthening attitudes towards team-oriented care. In Spain, health centres were established along with a team-based care concept that encompasses close physician-nurse collaboration and an autonomous role for nurses in the care process. Nurses collaborate with GPs on more equal terms with conflicts centring on professional disagreements. Team development structures and financial incentives encourage teams to generate their own strategies to improve teamwork. Clearly defined structures, shared visions of care and team development are important for implementing and maintaining a good collaboration. |
| Mattheys et al., 2017 [39] | Review | not specified | To synthesize the evidence on the impact of collaboration between physicians and nurses in primary care. | Physicians and nurses collaborated effective primary care | Nurses do have added value, physicians and nurses collaborated in blood pressure, patient satisfaction and hospitalization collaboration appeared not to be effective in Colorectal screening, hospital length of stay and health-related quality of life. Collaboration between physicians and nurses may have a positive impact on several patient outcomes and on a variety of pathologies. |
| Sangalei et al., 2017 [40] | Systematic review | not specified | to synthesize evidence on the experiences of health professionals regarding teamwork and interprofessional collaboration | HCPs’ experience teamwork and interprofessional collaboration in PHC | Health providers face enormous ideological, organizational, structural, and relational challenges while promoting teamwork and interprofessional collaboration in primary health care settings. Possible actions could improve implementation of teamwork and interprofessional collaboration in PHC. |
| Warmelink et al., 2017 [41] | Quantitative | The Netherlands | To provide insight into the professional working relations of primary care midwives. | Primary care midwives in collaboration of maternity care providers varies within care continuum. | Interactions with non-physicians are ranked consistently higher on satisfaction than physicians. Midwives with more work experience were more satisfied with their collaboration with GPs. Midwives were more satisfied with collaboration with GPs and obstetricians, the midwives were more satisfied regarding their collaboration with clinical midwives. Inter-professionals’ relations in maternity care can be enhanced the primary care midwives' interactions with physicians and with maternity care providers in urban areas. |
| Andrews et al., 2018 [42] | Literature review | US | To provide Comprehensive Care Workgroup of the Elimination of Perinatal HIV Transmission Stakeholders Group | Developed a concept of perinatal HIV service coordination (PHSC) | leadership strategies for implementing the core functions of PHSC included: strategic planning, access to services, real-time case finding, care coordination, comprehensive care, and data and case reviews. PHSC provides a systematic approach to optimize services and close gaps in perinatal HIV prevention and the HIV care continuum for childbearing women that can be individualized for jurisdictions with varying needs. |
| Blumenthal et al., 2018 [43] | Quantitative | US | To examine how team dynamics relate to perceptions of safety culture in primary care. | Relationship of team dynamics perception of safety culture | Care coordination mediated the relationship between team dynamics and the perception of safety culture, relationship between team dynamics, care coordination and perceptions of patient safety in a primary care setting. it needs to pay more attention to how primary care providers work together to coordinate care to make patient safer. |
| Valaitis et al., 2018 [44] | Qualitative | Canada | To identify the influencing factors within these organizations that affect the ability of these health care sectors. | organizational influencing factors on collaboration | Factors were Clear Mandates, Vision, and Goals; Strategic Coordination and Communication Mechanisms between Partners; Formal Organizational Leaders as Collaborative Champions; Collaborative Organizational Culture; Optimal Use of Resources; Optimal Use of Human Resources; and Collaborative Approaches to Programs and Services Delivery. Interactions among these influences are indicative of the complex nature of public health and primary care collaboration, and needs to set up new or maintain existing collaborations with public health and primary care which may or may not include other organization |
| Gyllstrom et al., 2019 [45] | Mixed methods | US | To describe the degree of public health and primary care collaboration at the local level | A framework of collaboration, the Community Collaboration Health Model (CCHM) | Wide variation in relationship factors including foundational characteristics, present in current working relationships but were less likely to agree that relationships had factors promoting sustainability or innovation. Identifying shared priorities and achieving tangible benefits may be critical to realizing sustained relationships resulting in population health improvement. Tools, such as the CCHM, and technical assistance may be helpful to support advancing collaboration.  Key components of promoting collaboration- Dedicated funding, reimbursement redesign, improved data systems, and data sharing capability |
| Hustoft et al., 2019 [46] | Quantitative | Norway | To investigate how changes in patient-rated health and disability from baseline to after rehabilitation. | Association of communication and relationships in rehabilitation teams and patient-rated continuity of care | Associations between continuity of care and changes in patient-rated health, communication was associated with more improvement in functioning in neoplasms patient group. Themes were better personal, team and cross-boundary continuity of rehabilitation care was associated with better patient health after rehabilitation. Measures of patient experiences with different types of continuity of care may provide a promising indicator of the quality of rehabilitation care. |
| Javanparast et al., 2019 [47] | Mixed methods | Australia | To examine the strength and extent of collaborations between PHC organisations and local government in population health planning. | Limited capacity of local organisation for collaboration to tackle social factors | Medicare Locals/ Primary Health Networks reported limited time and financial support for collaboration with local government. Organisational capacity and resources, supportive governance and public health legislation mandating a role for local governments were critical to collaborative planning. Local government has the potential to tackle social factors affecting health. Strategies could be the inclusion in population health planning, legislative mandates with stronger Federal Government mandate backed by sufficient resources and a governance structure to support collaboration. Improving PHC and local government collaboration has great potential to improve the quality of health planning and action on social determinants. |
| Kates et al., 2019 [48] | Qualitative | not specified | To build and present a global framework for enhancing mental health care delivered within primary care | Three pronged approaches of collaborative care in mental health | Primary care provider can deliver with or without the presence of a mental health professional; effective collaboration can enhance this care; at wider system changes required to support these new roles and how better collaboration can lead to new responses to respond to challenges facing all mental health systems. This framework can be applied in any country to enhance the detection, treatment, and prevention of mental health problems, reinforcing the role of the primary care provider in delivering care and showing how collaborative care can lead to better outcomes for people with mental health and addiction problems. |
| Stumm et al., 2019 [49] | Qualitative | Germany | To identify the barriers to the successful coordination of multimorbid patient care and these patients' complex needs, and to explore the support needed by GPs in the care of multimorbid patients. | Coordination in the context of care for multimorbid patients consists of a wide range of different tasks | Organisational and administrative obstacles under the regulatory framework, and insufficient communication with healthcare providers constitute barriers. GPs may have to delegate responsibilities associated with coordinating tasks and consider the deployment of an additional specifically qualified employee inside the general practice to take on coordinative and social and legal duties. The cooperation among all involved key players the coordination of the whole care process, is challenging for GPs within the complex care system of multimorbid patients. GPs are generally open to the assignment of a person to support them in coordination tasks, preferably situated within the practice team. |
| Witt et al., 2020 [50] | Qualitative | Australia | To explore health professionals' perspectives on communication, continuity, and between-service coordination for improving cancer care for Indigenous people. | Communication and collaboration in the provision of cancer care for indigenous populations | PHC sites of ACCHOSs were in urban, regional, and rural settings timely communication and information exchange, collaborative approaches, streamlined processes, flexible care delivery, and patient-centred care and support were crucial in improving the continuity and coordination of care between the PHC service and the treating hospital. Communication, collaboration, and care coordination are integral in the provision of quality cancer care for Indigenous Australians. Health policy and funding be designed to incorporate these aspects across services and settings as a strategy to improve cancer outcomes for Indigenous people. |
| Shoesmith et al., 2020 [51] | Qualitative | Malaysia | To better understand collaboration between and within systems and create a theoretical framework for system development. | Enabling collaboration | Collaborative behaviours; motivation towards a common goal or value; autonomy, relatedness (for example trusting, understanding, and caring about the other); resources (competence, time, physical resources, and opportunities); and motivation for collaboration (weighing up the personal costs versus benefits of acting collaboratively). barriers to collaboration were lack of autonomy, relatedness, motivation, and resources, together with the potential cost of acting collaboratively without reciprocation. change these structural, cultural, and organisational features could improve collaboration to access to care and outcomes for patients. |
| Fox et al., 2021 [52] | Qualitative | Canada | To investigate how the ideals of patient centeredness and clinical democracy put forward in the IP against actual IPC practice. | Studying actual communication practices to understand collaborative activities | interprofessional practices was created and compared to the continuum of interprofessional collaborative practice, analysed how participants made sense of their collaboration. Findings were grouped into three categories of communicative actions: coordinating sequential efforts; assisting others' sensemaking; and working to understand together. |
| Korstjens et al., 2021 [53] | Qualitative | The Netherlands | To understand how professionals and parents in maternity care accomplish constructive communication and collaboration, we examined their interactions in the clinic | Role of informal communication and collaboration in maternity care | Informal strategies facilitate communication and collaboration: “talk work” – small talk and humour – and “work beyond words” – familiarity, use of sight, touch, sound, and non-verbal gestures. While using information communication, context, values, feelings, and timing of care are important good communication and collaboration involves “paradoxical care”, e.g., concurrent acts of “regulated spontaneity” and “informal formalities”. Good communication and collaboration in maternity care involves “paradoxical care” requiring social sensitivity and self-reflection, skills that should be included as part of professional training. |
| Maheen et al., 2021 [54] | Mixed methods | Pakistan | To estimate the continuum of care utilisation rate and factors of women. | Low continuum of care during pregnancy | Limited knowledge about affordable health services, poor health literacy, and access to health services was associated with women's fragmented utilisation of maternity care. Barrier of utilisation of PHC included a lack of respectful maternity-care. The existing primary health structure in Pakistan provides a good foundation to deliver continuity of care services. Health services utilisation for reproductive and maternity care remains suboptimal in remote Pakistan. |
| Ramsden et al., 2021 [55] | Qualitative | Australia | To explore how four small towns in rural New South Wales in addressing challenges accessing quality care and sustainable health services | Collaborative care framework is a useful planning and community engagement tool | Collaborative care framework is important tool to build health workforce literacy and to impact on system change at the local level. Key elements included the need for coordinated health system planning, better integrating existing resources to deliver services, community engagement, building health workforce literacy and town-based planning. Findings add how to successfully develop a collaborative PHC workforce model in practice. The implementation of a collaborative PHC workforce can improve service access and quality and facilitate workforce sustainability. |
| Rawlinson et al., 2021 [56] | Review | not specified | To identify barriers and facilitators of IPC in primary care settings | Barriers and facilitators of interprofessional collaboration | Barriers- time and training, lack of clear roles, fears relating to professional identity and poor communication. facilitators of IPC- communication, co-location and recognition of other professionals' skills and contribution. these drivers go beyond specific local contexts and can prove useful for the development of tools or guidelines for successful implementation of IPC in primary care. |
| Seaton et al., 2021 [57] | Review | not specified | To explore the perceptions of allied health professionals regarding interprofessional collaboration in primary health care. | Key elements related to interprofessional collaboration in PHC | Themes of interprofessional collaboration included shared philosophy; communication and clinical interaction; physical environment; power and hierarchy; and financial considerations. Opportunity for frequent, informal communication appeared essential for interprofessional collaboration to occur. Allied health professionals working near health practitioners from other professions had more regular interprofessional interactions than those who were geographically separated. Co-location of multiple PHC services within the same physical space may offer increased opportunities for IP collaboration. |
| Silva et al., 2022 [58] | Quantitative | Brazil | to analyse the satisfaction of people with hypertension about the coordination of care in PHC. | People with hypertension had satisfaction with the coordination of care in PHC | Interviewees satisfactorily evaluated the use of medical records during consultation and results in the specialized service. While they unsatisfactorily evaluated the recording of complaints and health needs verbalized during consultations, scheduling of return visits, and written referrals, and guaranteed care and clarifying information about the results of the consultation in the referral service. |
| Gemmeke et al.,2022 [59] | Qualitative | Netherlands | To explore the perceptions of primary care providers on multidisciplinary collaboration in fall prevention | Limited collaboration in fall injury prevention | Allied health professionals reported to collaborate multidisciplinary to prevent falls. They limited collaboration with community pharmacists, had limited knowledge on drugs that increase the risk of falls, low awareness of the potential role of pharmacists in fall prevention. Reasons of poor collaboration included lack of agreements with pharmacists, limited coordination, and communication. Multidisciplinary agreements among health care providers, including community pharmacists, about referral criteria, roles and responsibilities, communication, and coordination, could stimulate further collaboration in fall prevention. |
| Lago et al., 2022 [60] | Qualitative | Brazil | To analyse the resistance to interprofessional collaboration in the professional practices of residents in PHC. | Contradiction of uni-professional education and interprofessional collaborative practices | There were contradictions between the reproduction of uni-professional education with a focus on the specialty and interprofessional collaborative practices with not-knowing as an analyser of resistance to collaboration; interprofessional interference and knowledge-power relations. Residents' practices were characterized as resistant to interprofessional collaboration. The Multi-professional Residency showed integrative movements of assimilation and disputes with physician-centered power, with damage to the sharing of care and interprofessional communication. |
| Vähätalo et al., 2022 [61] | Qualitative | Finland | To understand how professionals experience the cooperation between public PHC and occupational health services | Professional experiences of PHC and occupational health services | Three key themes were attitudes toward the other health care sector, the exchange of information, and resources for cooperation. Professionals seem to have poor knowledge about the services available and how care is given in the other sector, appearing to lead to weak mutual trust. The public PHC professionals emphasized the benefits of cooperation, but several issues were mentioned as barriers to cooperation. |
| Walton et al., 2022[62] | Qualitative | UK | To develop and evaluate strategies to potentially improve care coordination | Six domains of strategies to improve care coordination | Ways of organising care (local, hybrid, national), Ways of organising those involved in care (collaboration between many or all individuals, collaboration between some individuals, a lack of collaborative approach), Responsibility for coordination (administrative support, formal roles and responsibilities, supportive roles and no responsibility), How often appointments and coordination take place (regular, on demand, hybrid), Access to records (full or filtered access), and Mode of care coordination (face-to-face, digital, telephone). This may help to facilitate the development and evaluation of existing and new models of care coordination for people living with rare conditions. |
| Wierdsma et al., 2009 [63] | Qualitative | not specified | To describe conceptual consensus is growing, there is room to improve continuity measures, and the development of practical interventions | concept of COC- Continuity of care as ‘continuous care. Continuity of care as an ‘individual relationship ‘Continuity of care as a ‘seamless service’. Continuity of care ‘recorded objectively’ | Continuity of care is continuous care; care of an individual patient; cross-boundary care; and care recorded objectively. Continuity of care is informational continuity, management continuity, relational continuity and contact continuity. Factors hindering COC were the lack of standard measures and administrative data appropriate to assessing continuity. COC can be explained as logic model: structure (integrated care), processes (continuity of care) and outcome (quality of care) and levels of coordination: district level (informational continuity), provider level (management continuity), team level (relational continuity), patient level (contact continuity) |
| Lin et al.,2010 [64] | Quantitative | Taiwan | To determine whether the discontinuity of care is associated with the risk of hospitalization. | COC and avoidable hospitalisation | Risk of hospitalization increased due to patients with low to medium continuity of care. Higher COC with usual providers for diabetic care reduced risk of future hospitalization for long-term diabetic complication admissions |
| Lafferty et al., 2011 [65] | Qualitative | Northern Ireland | To explore 1) the concept of COC from the perspectives of women with breast cancer, their careers, and their HCPs | COC in breast cancer | ‘COC’ is the continuous care over time; it involves the relationship between the HCP and the patient. The relationship is built on trust, loyalty, and constancy. |
| Rabkin et al., 2012 [66] | Qualitative | LMICs | To strengthen health systems to support services for noncommunicable diseases in LICs | COC in multiple diseases | COC was critical for older PLWH who risk for rapid progression of disease were, complication requires treatment, they have had chronic NCDs (hypertension, diabetes, cancers). Chronic care systems, lessons and resources can also be leveraged to support the people with HIV-negative but with chronic NCDs. |
| Shade et al., 2012 [67] | Quantitative | not specified | To describe how comprehensive HIV care is delivered within Ryan White Program funded clinics and to characterize proposed health information exchange (HIE) interventions | Health information for COC in HIV care | There as declining coverage cascade in HIV treatment. COC requires health information exchange (HIE) interventions to access a variety of data systems (e.g., surveillance, electronic health records, laboratory, and billing) and focused on improving linkage and retention, quality and efficiency of care and increased access to patient information |
| Sweeney et al., 2012 [68] | Quantitative | UK | To explore a new construct of service user defined COC and its relationship to a range of health and social outcomes. | Users’ continuity of care in health and social care | Service user-defined COC is underpinned by three measurable sub-constructs: preconditions, staff-related continuity, and care contacts. Preconditions for COC were symptoms and quality of life. COC was unrelated to empowerment and care contacts unrelated to outcomes. Service users with hospital admission experienced high COC. |
| Banfield et al.,2013 [69] | Qualitative | Australia | To 1) explore how information continuity supports coordination and 2) investigate conditions required to support information continuity. | Coordination of care in patient centered care | Accessibility and continuity of information underpin effective care but are insufficient for COC for complex conditions. Shared information reduced unnecessary repetition and provided HCPs with the opportunity to access records of care. COC increased users’ engagement in person care coordination than passive availability of information. Data ownership and confidentiality hampered information sharing. Coordination was associated with responsiveness. For the patient or coordination for system, there was insufficient availability of information to ensure continuity |
| Wook et al., 2014 [70] | Qualitative | South Korea | To determine the impact of COC on health outcomes in patients with CVD. | Continuity of Care on Mortality and Health Care Costs | Low COC was also associated with increased inpatient and outpatient days and costs in CVDs |
| Fernández et al., [71] | Qualitative | Argentina | To redesign the Contingency Plan Procedure. | Electronic Health Record System Contingency Plan Coordination | Effective communications channel, formal structure for functions support decision making were strategies for redesign the contingency plan and strategic actions. The actions included incorporation of IT contingencies in supervision and redefining clinical information |
| Waibel et al., 2015[72] | Qualitative | Sweden | To analyse the COPD patients’ perceptions of continuity of clinical management and information across care levels and continuity of relation in IHN of the public health care system. | performance of integrated health care networks in continuity of care.  continuity of clinical management  continuity of information.  Continuity of relation | In chronic diseases (COPD), COC included continuity of clinical management (distribution of roles for COPD care across levels, rapid access to care, referrals to secondary care), continuity of information (transferred across levels via computer and that physician also used informal communication mechanisms) and continuity of relation (patients of networks and trust). Challenges of COC: long waiting times, unclear distribution of roles, inadequate referrals, staff turnover |
| Ye et al.,[73] | Quantitative | China | To evaluate the effect of COC in a cohort study among hypertensive patients aged >35 years | continuity of care on health-related quality of life in adult patients with hypertension | Patient with high continuity of care group presented greater improvement in physical and mental component physical functioning, role-physical, general health, role-emotional, and mental health than low continuity of care group |
| Vargas et al., 2017 [74] | Quantitative | Brazil | To analyse the level of continuity of health care perceived by users and explores influencing factors in two municipalities of Colombia and Brazil, | Lower levels of continuity of information and care coherence across health care levels. | Levels of continuity across care was low in information transfer, care coherence, but was high in ongoing patient-doctor relationship. Factors of COCs were consistency of doctors and health needs. Factors consistency of doctors, study area and declared morbidity.  Addressing discontinuities requires effective policies to improve coordination across care levels |
| Meyer et al., 2018[75] | Mixed methods | south Africa | To understand how COPC with CHWs visiting households offering health education can support ANC follow-up and the barriers for access to care | Each needs high touch: Supporting continuity of community primary health care | An integrated COPC approach increased follow up and tracking of pregnant women at home. Women was afraid of denied care so gave wrong addresses or personal identification. Mobilisation of ward-based outreach team was challenging due to lack of patent residence. |
| Chukwuma et al.,2019 [76] | Mixed methods | Tajikistan | To undertake a systematic assessment of hypertension case detection and retention in care within Tajikistan’s PHC system | continuity of care in HTN | Gaps and berries of case detection stage of the cascade of care were misinformation about hypertension, ambiguous protocols, and limited delivery capacity. Solutions were mobilizing faith-based organizations, scaling up screening through health caravans, task-shifting to increase provider supply, and introducing job aids for providers |
| Wylie et al., 2020 [77] | Qualitative | Canada | To explore the system-level issues that affect the access to, and quality and outcomes of mental health care for immigrants and refugees, with a particular focus on challenges in the continuity of patient care. | Strengthening continuity of patient care to improve the mental health of immigrants and refugees | A multidisciplinary group of HCPs identified six themes: perceived access to care, coordination amongst HCPs, patient connections with community organizations; coordinated care planning, organizational protocols, policies and procedures and systemic and health care training needs. Creating a resilient health care system is vital for anticipate and adapt to adverse situations. Advocating for public health policy creates systemic resilience for the continuity of health care |
| Khatri et al., 2021 [78] | Quantitative | Nepal | examined the levels and correlates of (dis)continuity of maternity care across the antenatal-postnatal period. | continuity of care along antenatal through to PNC pathway | Low completion of (41%) of maternity visits with high discontinuation around childbirth. Women had high risk of discontinuation if they were from disadvantaged ethnicities, from low wealth status, who were illiterate, living in remote areas, had poor awareness of health mother-groups, and not have female provider |
| Helmyati et al., 2022 [79] | Mixed methods | Indonesia | To implement an online system to evaluate the impact of the covid-19 pandemic on MCHN essential health services. | continuity of maternity care | Pandemic affected key MCHN services (child growth monitoring and ANC), such as adherence to the protocol. The health institutions and academic partners provided the platform for collaborative efforts for health-systems strengthening. The electronic monitoring and evaluation system implemented and completed several modifications to accommodate |
| Susanti et al., 2022 [80] | Mixed methods | Indonesia | To identify and explore midwives’ competency and service needs to develop mHealth in Midwifery Continuity of Care (MCOC) education and training. | Midwifery Continuity of Care in Indonesia: Initiation of Mobile Health Development Integrating Midwives’ Competency and Service Needs | Most midwives needed to develop competency in the MCOC scope (its early detection of the risk factor of complications and treatment management) including the purposes, benefits, and design of mHealth. in MCOC, Midwives’ competency indicators using mHealth are used for early detection |
